# Measurement and determinants of catastrophic health expenditure among elderly households in China using longitudinal data from the CHARLS

**DOI:** 10.1186/s12939-020-01336-8

**Published:** 2021-02-19

**Authors:** Shiai Liu, Peter C. Coyte, Mingqi Fu, Qilin Zhang

**Affiliations:** 1 Center for Social Security Studies of Wuhan University, Wuhan, Hubei China; 2 Institute of Health Policy, Management and Evaluation, University of Toronto, Toronto, Canada

**Keywords:** Catastrophic health expenditure, Determinants, Elderly households, China

## Abstract

**Background:**

Catastrophic health expenditure (CHE) among the Chinese elderly warrants attention. However, the incidence, intensity and determinants of CHE have not been fully investigated. This study explores the incidence, intensity and determinants of CHE among elderly Chinese citizens, i.e., those aged 60 years or older.

**Methods:**

Data were obtained from three waves of the China Health and Retirement Longitudinal Study (CHARLS): 2011, 2013 and 2015. The cut-off points used in this study for CHE were 10% of the total expenditures and 40% of non-food expenditure. Under the guidance of Andersen’s model of health services utilization, this study used logistic regression analysis to explore the determinants of CHE.

**Results:**

The incidence of CHE defined as more than 40% of non-food expenditure rose over the study period, 2011–2015, from 20.86% (95% CI: 19.35 to 22.37%) to 31.00% (95% CI: 29.28 to 32.72%). The intensity of CHE also increased. The overshoot (O) based on non-food expenditure rose from 3.12% (95% CI: 2.71 to 3.53%) to 8.75% (95% CI: 8.14 to 9.36%), while the mean positive overshoot (MPO) rose from 14.96% (95% CI: 12.99 to 16.92%) to 28.23% (95% CI: 26.26 to 30.19%). Thus, the problem of CEH was even more serious in 2015 than in 2011. Logistic regression revealed that households were more likely to face CHE if they had a spouse as a household member, reported an inpatient event in the last year, reported an outpatient visit in the last month, were disabled, were members of a poor expenditure quartile, lived in the middle and western zones or resided in an urban area. In contrast, CEH was not significantly affected by respondents being older than 75 years or having a chronic health condition, by household size or by insurance type.

**Conclusions:**

Key policy recommendations include the gradual improvement of medical assistance and the expansion of the use of health insurance to reduce household liability for health expenditures.

## Background

Health disorders are associated with large economic burdens on individuals as well as households. For individuals with serious health conditions and limited financial resources, exposure to high medical expenses may move the household into debt [1]. On occasions, this debt may be a burden over the remaining course of a person’s life. Worldwide, approximately 150 million individuals are reported to live with severe financial difficulties due to large health expenditures, with over 60% of these persons residing in low-middle income countries [2]. As the largest low-middle income country in the world, China faces serious challenges in dealing with catastrophic health expenditure (CHE). In 2015, the prevalence of poverty associated with onerous health expenditures was high, at 44.1% [3]. Therefore, it is necessary to further deepen the research on this topic to effectively resolve and respond to the poverty problem brought about by the economic risk of disease.

China officially launched the New Health Care Reform (NHCR) in 2009 with an overarching aim of reducing the financial burden of health expenditures on households. Under the NHCR, universal health insurance was expanded to cover both urban and rural residents. By 2017, two separate health insurance arrangements ensured universal coverage for all Chinese residents: the UEBMI (the Urban Employee Basic Medical Insurance) scheme, which was designed for those employed in (and retired from) the formal sectors, and the UBMI (Unified Basic Medical Insurance) scheme, which is available to all rural residents and those urban residents without formal employment. With the implementation of these insurance arrangements, the demand for medical services has grown dramatically, but the consequences for CHE have yet to be explored in detail, as many out-of-pocket medical expenses exist. Therefore, whether household CHE has been reduced still needs to be discussed in depth.

Of all groups in society, the elderly aged 60 years and older are at the greatest risk of incurring high health care expenses. In 2011, this group accounted for approximately 13.7% of the total population. The fifth National Health Service Survey (NHSS) of China, conducted in 2013, demonstrated that the outpatient visit ratio over 2 weeks among elderly people was 56.9%, the prevalence rate of chronic diseases was as high as 71.8%, and the annual rate of inpatient visits was 17.9% [4]. These figures were much higher than those recorded for other groups. Paired with this high demand for health care services among the elderly is their limited income, leading to a rise in exposure to high health care expenses. International evidence has shown that people of lower economic status are more likely to suffer from serious illness and become impoverished due to health care expenses [5]. Of even greater concern is the rapid ageing of the population, which will have a major impact on future health care costs for the elderly and their households and for society. According to the “National Population Ageing Development Trend Forecast Research Report” issued by the National Committee on Aging in 2015, the number of elderly Chinese residents will reach 437 million by 2051, at which time, this group will account for 30% of the total population.

Indeed, inquiry into catastrophic health expenditure (CHE) has become a hot issue in health studies in China. CHE in rural areas has been discussed extensively in the literature [3, 6, 7]. Some scholars have studied CHE among patients with chronic diseases [8, 9] and migrants [10, 11]. Although several studies have examined the prevalence of CHE in China, no consensus has been reached to date, as each study has used different databases and methodologies. One study based on data from the fourth NHSS suggested that the prevalence rate of CHE was 13.0% [12], while another study found that among elderly rural residents, it was 25.6% [13]. Existing studies that have analysed CHE in different age groups report that it varies by age. Generally, households with members over 65 years of age and under 5 years of age are more vulnerable to CHE [14], and the proportion of CHE in elderly households is 3.71 times that for non-elderly households [15]. Additionally, the determinants of CHE have been extensively explored in previous studies. Household economic status, the inpatient rate, the presence of an elderly or disabled household member, and the presence of a household member with chronic illness were commonly associated with CHE [16–18]. While it was thought that health insurance would help to alleviate some of the economic burden brought about by disease, the evidence has remained unclear. Some scholars believe that health insurance has been helpful in reducing the prevalence of CHE [19], while others report either no effect [20–22] or limited effects [7, 18, 23]. A recent study has analysed the mechanism behind the multilevel medical security that reduces CHE [24].

While earlier studies highlight the importance of CHE in China, those studies have several limitations. First, there has been very little focus, to date, on the elderly in China. To our knowledge, only one paper has focused on rural elderly Chinese individuals [21], but the data used in that paper were quite dated (i.e., before 2011) and were not longitudinal, and the study suffered from potential heterogeneity bias. Second, the impact of the various health insurance schemes in China on CHE remains unclear. Third, previous studies have varied in their choice of influencing factors and have not been guided by an explicit conceptual framework that would assist in variable identification and in the specification of the data-generating process.

The purposes of this study are threefold: to measure trends in the incidence and intensity of CHE among elderly Chinese aged 60 years or older from 2011 to 2015 using three waves of the CHARLS; to identify the factors that account for variations in the incidence of CHE by referring to Andersen’s model of health services utilization, with special attention to the role played by different health insurance schemes; and finally, to describe more precise and evidence-based measures that reduce the prevalence of CHE among the elderly in China.

## Methods

### Data sources

The study sample was drawn from three waves (2011, 2013 and 2015) of the China Health and Retirement Longitudinal Study (CHARLS). The CHARLS was implemented by the National Development Research Institute of Peking University. It collected information on a range of variables, including demographic background, family structure, work status, retirement and pension status, household expenditures, and health information (health status, insurance coverage, and health service utilization).

**Fig. 1:**
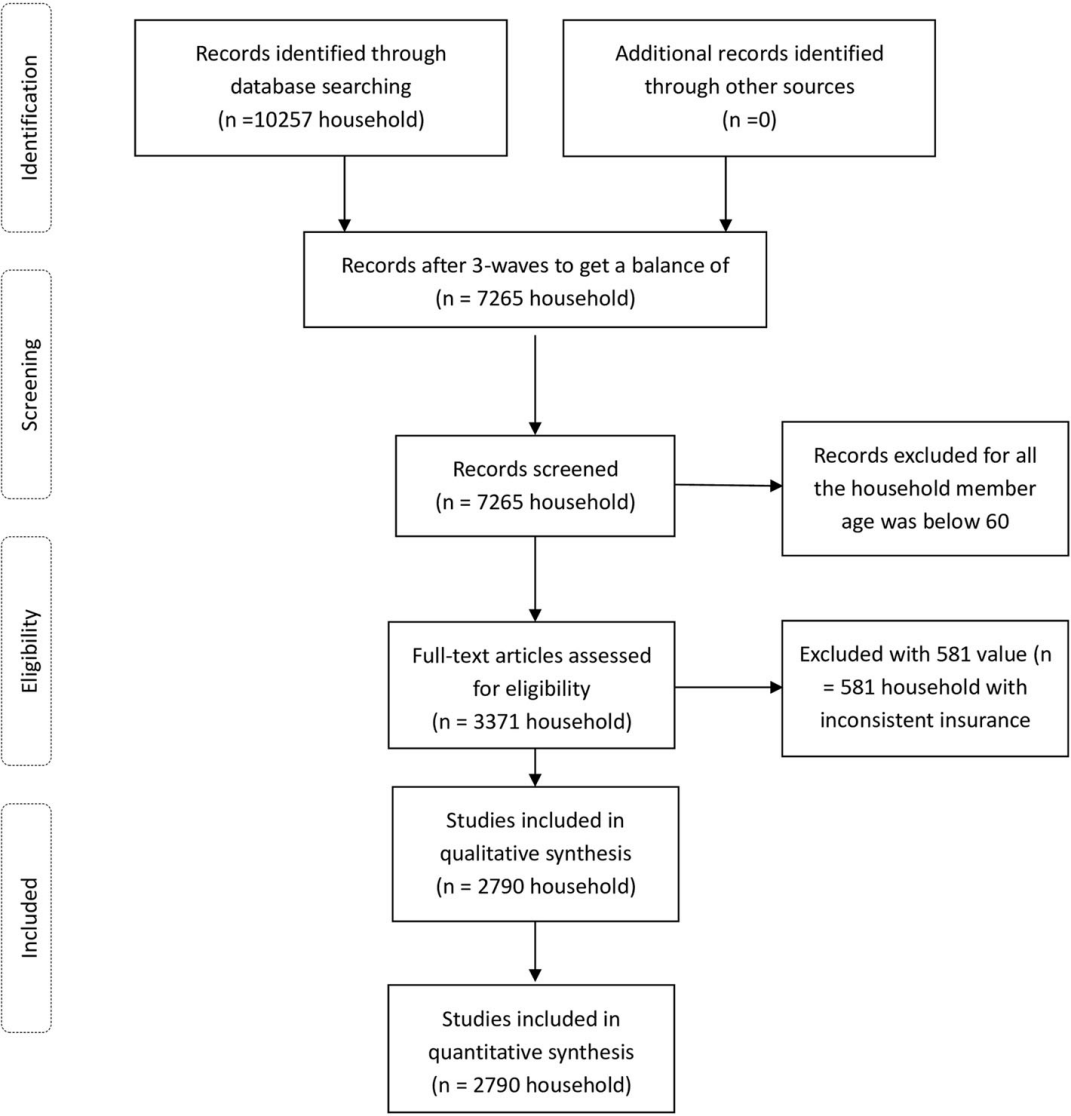
Procedure for participants selection

The baseline survey was conducted between June 2011 and March 2012. Household members aged 45 years or older were invited to participate in this survey, and his/her spouse became a respondent automatically. A total of 12,740 households participated in the survey, for a response rate of 80.51%. Data from 10,257 households were eventually retained (Fig. 1). These households were re-surveyed every 2 years; however, for many reasons, such as migration or death, there were only 3371 households in all three survey waves for all individuals aged 60 years or older, and we deleted 581 households with inconsistent insurance. Finally, a total of 2790 households were included in our analysis in this study.

### Specification of the empirical model

This paper uses a binary choice model for panel data, takes the occurrence of CHE as the dependent variable, and takes Andersen’s medical services utilization model as the theoretical basis. The binary choice model for CHE panel data can be constructed as follows:

1 $$ {y}_{it}^{\ast }={\boldsymbol{x}}_{\boldsymbol{it}}\boldsymbol{\beta} +{\mu}_i+{\varepsilon}_{it}\ \left(i=1,\dots, n;t=1,\dots, T\right) $$

where ***x***_***it***_ is the set of explanatory variables; **β** is the parameter vector corresponding to the explanatory variables; *μ*_*i*_ is the individual item; *ε*_*it*_ is the residual item estimated by the model; and ${y}_{it}^{\ast }$ is the unobservable variable (latent variable). When ${y}_{it}^{\ast }$ > 0, it is recorded as 1; otherwise, it is recorded as 0. Given ***x***_***it***_, **β**, *μ*_*i*_:

2 $$ P\left({y}_{it}=1|{x}_{it},\boldsymbol{\beta}, {\mu}_i\right)=P\left({y}_{it}>0|{x}_{it},\boldsymbol{\beta}, {\mu}_i\right)=F\left({\mu}_i+{\boldsymbol{x}}_{\boldsymbol{it}}\boldsymbol{\beta} \right) $$

where *F*(•) is the cumulative distribution function of *ε*_*it*_. Assuming it obeys the logistic distribution, it is the logit model:

3 $$ P\left({y}_{it}=1|{x}_{it},\boldsymbol{\beta}, {\mu}_i\right)=\varLambda \left({\mu}_i+{x}_{it}^{\prime}\boldsymbol{\beta} \right)=\frac{e^{u_i+{x}_{it}^{\prime}\boldsymbol{\beta}}}{1+{e}^{u_i+{x}_{it}^{\prime}\boldsymbol{\beta}}} $$

There are three main types of binary selection models, pooled, random effects and fixed effects logit models, for use with panel data [25]. Based on the results of the Hausman specification test, we selected the fixed effects logit method for our baseline results. All data analyses were performed using STATA 15.0 (StataCorp LP, College Station, Texas).

### Dependent variable

CHE is defined as household health care expenditures that exceed certain fractions of household total income or non-food expenditure [5, 26]. However, in low- and middle-income countries, income information is frequently unavailable or of poor quality [27]. Consumption expenditure is often preferred to income as a measure of socioeconomic status due to more accurate reporting [28]. As yet, there is no consensus on the threshold value to apply to define CHE. Generally, two thresholds are widely used: i) out-of-pocket healthcare payments (OOP) that comprise more than 10% of total expenditure [29, 30] or ii) out-of-pocket healthcare payments that comprise more than 40% of non-food expenditure [26, 31]. The health expenses of the surveyed households include only the respondent and his/her spouse. The total household expenses and food expenses are extracted from the questionnaire.

CHE is usually assessed in terms of incidence and intensity. Headcount (*Hc*) is used to measure incidence, while overshoot (*O*) and mean positive overshoot (*MPO*) are used to measure intensity. HC is the percentage of households whose OOP payments as a proportion of their total income or non-food expenditure equal or exceed a certain threshold. Overshoot (O) is the average amount by which payments equal or exceed certain fractions for all the sample households, while mean positive overshoot (MPO) is the average amount for those whose household experienced CHE [29].

In this study, CHE = 1 if health expenditure was compared with total expenditure (≥10%) or with net non-food expenditure (≥40%), and CHE = 0 otherwise [29, 31].

### Independent variables

The independent variables were associated with Andersen’s model of health services utilization. In this model, the variables that determine utilization were categorized into predisposing factors (age, sex, ethnicity, marital status, etc.), enabling factors (zone, area, health insurance type, household size, household socioeconomic status, etc.), and needs-based factors (perceived severity of illness, presence of physician diagnosing chronic diseases, etc.) [32].

Based on the CHARLS survey questionnaire, we chose age and marital status as predisposing factors. Because our unit of analysis is the household, we also use whether a household has members older than 75 years and whether the household has spouses living together or not as predisposing factors according to the survey questionnaire.

For the enabling factors, we chose zone, area, health insurance type, household size and household socioeconomic status. The zone was categorized as eastern, middle and western. The area categories were urban and rural. The urban area included households living in towns and urban neighborhoods in cities, while the rural area included households living in villages and suburban areas of cities. Health insurance was divided into four types. While UEBMI and UBMI cover almost all citizens, we included a category for other insurance (OI) to account for those without UEBMI or UBMI but with commercial medical insurance and/or other types of medical insurance; we also included a category for those without any medical insurance (NI). The household socio-economic status was divided into five economic status categories according to household aggregate expenditure (excluding health expenditure) from low to high, indicating the poorest, poorer, average, richer, and richest.

For the needs-based factors, we chose whether a household member with chronic diseases was diagnosed by a physician, whether a household member was disabled, whether a household member used outpatient services in the last month and whether impatient services were used in the last year.

## Results

### Descriptive statistics

Table 1 presents the descriptive statistics for each of the three waves of the CHARLS. The percentage of household members above 75 years of age was 16% in 2011, 23% in 2013 and 30% in 2015, and nearly 65 to 70% of the household members were living with a spouse. By 2015, 1.15% of respondents had no insurance, with UEBMI and UBMI accounting for 13.69 and 84.91% of the respondents, respectively. Over half (67%) of the households were in rural areas. Of the households, 25.59, 34.87 and 39.53% lived in the eastern, middle and western zones, respectively. For the needs-based factors, the inpatient rate of the sample was 15, 22 and 26% in 2011, 2013 and 2015, respectively. Over the study period, the overwhelming majority of households (83%) reported having a chronic condition and at least 30% of households had a disabled member. These summary data are consistent with the results of the fifth NHSS in 2013 [4].

**Table 1:** Household descriptive statistics^a^ (*N* = 2790)

Variables	Definition	2011	2013	2015
		Mean (SD^b^)	Mean (SD)	Mean (SD)
Health expenditure per month	Continuous variable	34 (109)	62 (273)	77 (301)
Total expenditure	Continuous variable	314 (357)	404 (693)	448 (709)
Non-food expenditure	Continuous variable	144 (219)	213 (518)	250 (593)
**Predisposing factors**				
Household member age above 75		0.16 (0.36)	0.23 (0.42)	0.30 (0.46)
Marriage	0 = No spouse 1 = With spouse	0.70 (0.46)	0.65 (0.47)	0.65 (0.48)
**Enabling factors**				
Household size	Continuous variable	2.54 (1.83)	4.28 (1.58)	2.38 (1.20)
Economic status	1 = Poorest 2 = Poor 3 = Average 4 = Richer 5 = Richest	2.57 (1.39)	2.50 (1.36)	2.37 (1.35)
Insurance type^c^	0 = NI 1 = UEBMI 2 = UBMI 3 = OI	1.81 (0.51)	1.80 (0.49)	1.84 (0.40)
Area	0 = Urban 1 = Rural	0.67 (0.49)	0.67 (0.47)	0.67 (0.47)
Zone	0 = Eastern 1 = Middle 2 = Western	1.14 (0.80)	1.14 (0.80)	1.14 (0.80)
**Need factors**				
Inpatient or not	0 = No 1 = Yes	0.15 (0.36)	0.22 (0.36)	0.26 (0.44)
Chronic disease or not	0 = No 1 = Yes	0.83 (0.37)	0.83 (0.38)	0.87 (0.34)
Disability or not	0 = No 1 = Yes	0.30 (0.46)	0.45 (0.50)	0.46 (0.50)
Outpatient or not	0 = No 1 = Yes	0.30 (0.46)	0.32 (0.47)	0.30 (0.46)

Note: (a) Sorting was performed according to the CHARLS survey data; (b) The standard deviation is shown in parentheses; (c) Abbreviations: NI, no insurance; UEBMI, Urban Employee Basic Medical Insurance; UBMI, Unified Basic Medical Insurance for urban residents without formal employment and rural residents; OI, other insurance

### Incidence and intensity of catastrophic health expenditure (CHE)

Table 2 summarizes the incidence (*Hc*) and intensity (*O* and *MPO*) of CHE for the 3 survey years. The results were calculated using the commonly recommended cut-off points of 10 and 40% associated with total and non-food expenditure, respectively [29, 31].

**Table 2:** Incidence and intensity of CHE among elderly households in China, from 2011 to 2015^a^

Year	2011	2013	2015
Out-of-pocket health care spending as a share of total expenditure (cut-off point =10%)			
Head count (SE)	29.92 (0.008)	36.52 (0.007)	39.42 (0.009)
*p*-value^b^		< 0.001	< 0.001
Overshoot (SE)	6.85 (0.003)	9.87 (0.004)	11.45 (0.003)
*p*-value^b^		< 0.001	< 0.001
Mean positive overshoot	22.89	27.03	31.25
Out-of-pocket health care spending as a share of non-food expenditure (cut-off point = 40%)			
Head count (SE)	20.86 (0.007)	27.63 (0.008)	31.00 (0.009)
*p*-value^b^		< 0.001	< 0.001
Overshoot (SE)	3.12 (0.002)	5.06 (0.002)	8.75 (0.003)
*p*-value^b^		< 0.001	< 0.001
Mean positive overshoot	14.96	18.31	28.23

Note: (a) Presented as % unless otherwise indicated; (b) Statistical testing was conducted by comparing the year-specific “head count” with the equivalent value in 2011

The incidence of CHE (accounting for > 10% of total expenditure) continued to rise during the study period, from 29.92% (95% CI: 28.23 to 31.63%) in 2011 to 39.43% (95% CI: 37.61 to 41.24%) in 2015. In contrast, when the incidence of CHE was defined as health expenditure exceeding 40% of non-food expenditure, the incidence of CHE also continued to rise during the whole study period, from 20.86% (95% CI: 19.35 to 22.37%) in 2011 to 31.00% (95% CI: 29.28 to 32.72%) in 2015.

When intensity of CHE was assessed as the value of the overshoot, regardless of which measurement method was adopted, we found that the intensity of CHE also increased. When based on total household expenditure for the whole study period, the overshoot (*O*) increased from 6.85% (95% CI: 6.27 to 7.45%) in 2011 to 11.45% (95% CI: 10.67 to 12.23%) in 2015. When based on household non-food expenditure, the overshoot (*O*) also increased, from 3.12% (95% CI: 2.71 to 3.53%) in 2011 to 8.75% (95% CI: 8.14 to 9.36%) in 2015.

In the case of the mean positive overshoot (*MPO*), it was interesting to note that in 2011, those spending more than 10% of their total expenditure on healthcare spent on average 32.89% (10% + 22.89%) of their total expenditure on healthcare. This proportion grew over the study period and reached 41.25% (10 + 31.25%) by 2015. When healthcare expenditure was considered as a share of non-food expenditure, the level of this mean positive overshoot was very similar. In 2011, those spending more than 40% of their non-food expenditure on healthcare spent on average 54.96% (40% + 14.96%) of their non-food expenditure on healthcare, and this grew to 68.23% (40 + 28.23%) by 2015. Consequently, the intensity of CHE grew over the study period.

### Determinants of CHE

Table 3 presents the results of the logistic regression analysis of the longitudinal data and the cross-sectional data from the 2015 CHARLS wave based on two different denominators: the determinants of CHE at 10% of total expenditure and the determinants of CHE at 40% of non-food expenditure. For the fixed effects model, variables such as gender and region were automatically deleted because they did not change over the survey period. We use the cross-sectional data from the 2015 CHARLS wave to analyse the impact of zone and area separately.

**Table 3:** Determinants of the prevalence of catastrophic health expenditure using a panel logistic regression model

	(1)^c^	(2)^d^	(3)^e^
Variables	Odds Ratio	Odds Ratio	Odds Ratio
**Predisposing factors**			
Household has a member older than 75 years (compared with younger than 75)			
Household has a member older than 75 years	1.370^a^ (0.107)^e^	1.162 (0.438)	1.061 (0.552)
Married (compare with no spouse)			
Living with spouse	1.731*** (0.004)	1.776*** (0.003)	1.767*** (< 0.001)
**Enabling factors**			
Zone (compared with eastern area)			
Middle area			1.363*** (0.008)
Western area			1.317** (0.017)
Area (compared with urban)			
Rural			0.825* (0.078)
Insurance status (compared with no insurance^b^)			
UEBMI	1.010 (0.982)	1.955 (0.106)	0.791 (0.592)
UBMI	1.305 (0.353)	1.503 (0.166)	0.664 (0.323)
OI	2.498 (0.214)	2.199 (0.270)	0.249 (0.216)
Economic status (compared with poorest)			
Poor	0.502*** (< 0.001)	0.583*** (< 0.001)	0.527*** (< 0.001)
Average	0.345*** (< 0.001)	0.449*** (< 0.001)	0.351*** (< 0.001)
Richer	0.198** (< 0.001)	0.327*** (< 0.001)	0.310*** (< 0.001)
Richest	0.079*** (< 0.001)	0.188*** (< 0.001)	0.116*** (< 0.001)
Household size (compared with fewer than 4 members)			
Household size more than 4	0.970 (0.763)	0.871 (0.179)	0.899 (0.596)
**Need factors**			
Impatient compared with not impatient			
Inpatient	1.793*** (< 0.001)	1.763*** (< 0.001)	2.641*** (< 0.001)
Chronic disease compared with no chronic disease			
Chronic disease	1.043 (0.910)	1.191 (0.626)	2.056*** (< 0.001)
Disability compared with no disability			
Disability	1.926*** (0.005)	2.424*** (< 0.001)	1.127 (0.189)
Outpatient compared with not outpatient			
Outpatient	2.706*** (< 0.001)	2.424*** (< 0.001)	3.294*** (< 0.001)

Note: (a) * *p* < 0.1, ** *p* < 0.05, *** *p* < 0.01; (b) Abbreviations: UEBMI, Urban Employee Basic Medical Insurance; NI, no insurance; UBMI, Unified Basic Medical Insurance for urban residents without formal employment and rural residents; OI, other insurance; (c) CHE was defined as 10% of total expenditure; (d) CHE was defined based on 40% of non-food expenditure; (e) The cross-sectional 2015 CHARLS data for logistic regression, and CHE was defined as 40% of total non-food expenditure; (f) *P*-value in parentheses

In column (1), where CHE was defined as 10% of total expenditure, age and household size were not significant determinants of CHE. Compared with not living with a spouse, living with a spouse increased the prevalence of CHE by approximately 1.73 times (*p* = 0.004). Compared with those with NI, who had to pay the total cost of health care services out-of-pocket, those with UBMI, UBMI and OI were estimated to be 1.01 (95% CI: 0.42 to 2.41; *p* = 0.982), 1.31 (95% CI: 0.74 to 2.29; *p* = 0.353) and 2.49 (95% CI: 0.59 to 10.57; *p* = 0.214) times more likely to experience CHE, respectively, though none of the differences were statistically significant. This means that the various types of health insurance did not significantly reduce CHE. The socioeconomic status of households was another key driver of CHE. Compared with the poorest group, the richest group was 0.08 (95% CI: 0.04 to 0.13; *p* < 0.001) times and the medium and the richer groups were 0.35 (95% CI: 0.25 to 0.47; p < 0.001) and 0.20 times (95% CI: 0.14 to 0.28; p < 0.001), respectively, more likely to experience CHE. The poor group was 0.50 times (95% CI: 0.39 to 0.64; *p* < 0.001) more likely than the poorest group to experience CHE. Regarding the need factors, those who used inpatient services in the last review year and those who used outpatient services in the last review month were 1.79 (95% CI: 1.42 to 2.27; p < 0.001) and 2.25 (95% CI: 2.19 to 3.34; p < 0.001) times more likely than those who did not use such services to experience CHE. Those whose households had member(s) with a disability were 1.93 (95% CI: 1.22 to 3.04; *p* = 0.005) times more likely to experience CHE than households with no disabled persons. Last, households with a member with a chronic disease were only 1.04 (95% CI: 0.51 to 2.15; *p* = 0.91) times more likely than households without a member with chronic disease to experience CHE; this difference was not significant.

In column (2), where CHE was defined based on 40% of non-food expenditure, most of the results were similar, though the effect of socioeconomic status was slightly larger than that in column (1). For example, the richest group was 0.19 times (95% CI: 0.12 to 0.30; *p* < 0.001) more likely than the poorest to experience CHE, while the average and the richer groups were 0.45 (95% CI: 0.33 to 0.61; p < 0.001) and 0.33 (95% CI: 0.23 to 0.47; p < 0.001) times more likely, respectively. The poor group was 0.58 (95% CI: 0.45 to 0.75; p < 0.001) times more likely than the poorest group to experience CHE.

In column (3), we use the 2015 CHARLS cross-sectional data to further examine the impact of zone and area on CHE. Here, we select 40% of non-food expenditure as the standard for CHE. The results reveal that households in the middle and western zones face a 1.36 (95% CI: 1.08 to 1.71; *p* = 0.008) and 1.32 (95% CI: 1.05 to 1.65; *p* = 0.017) times higher prevalence of CHE, respectively, than households in the eastern zone. Households in rural areas face a lower prevalence of CHE, approximately 0.83 (95% CI: 0.67 to 1.02; *p* = 0.078) times that of households in urban areas. The results for the other variables were very similar.

## Discussion

In this study, we estimated the overall incidence and intensity of CHE over 5 years among the elderly in China with longitudinal data from the CHARLS. We also explored the determinants associated with CHE. Our study has three important findings.

First, we observed that the incidence and intensity of CHE rose over the study period based on two measurement standards. A previous study reported that the probability of CHE could be higher than 50% for low-income rural households in China [18]. This was unexpected because many policies were adopted in this period to reduce the level of CHE. For instance, in 2012, China’s central government launched a catastrophic medical insurance (CMI) program to prevent people from being reduced to poverty by healthcare costs [33]. The financial subsidy for health insurance under UBMI increased from 31.32 to 61.01 US dollars. It is therefore difficult to explain why the incidence and intensity of CHE continued to rise. One potential explanation is that in this period, health care expenditure increased significantly (our findings indicate that both the mean and median costs tripled in this period). In China, the main payment method for hospital charges is fee-for-service. In the absence of effective expenditure controls and with limited risk-sharing by the hospital, financial risk has been shifted to patients [34]. Hospitals have few incentives to control costs in a profit-seeking environment.

Second, the study showed that social health insurance programs have neither reduced the risk of catastrophic spending nor relieved the financial burden of the elderly in China. A study in China showed that health insurance increases health care usage, and as a consequence, the risk of CHE may also increase [35]. We also found similar CHE levels between the UEBMI and UBMI groups. When OOP payments for health care were high, those with UBMI may have chosen not to seek medical care to avoid becoming impoverished by health expenditures because reimbursement under UBMI is lower than that under UEBMI. In contrast, those who had UEBMI had few financial concerns regarding access to medical care and used many medical services but could still experience CHE. This finding indicates that the skill of managing health insurance to control health expenses should be further improved.

Third, our logistical regression results show that healthcare needs and service utilization are key determinants of catastrophic health expenditure. These results agreed with those of previous studies [14, 36–38]. Our study found that at least 83% of households had member(s) with chronic disease. Our study also found that the risk of CHE was closely linked with economic status. Overall, there was a pro-poverty effect among the elderly: the poorer the elderly were, the more likely they were to suffer CHE.

Our research has important documentary value for recognizing the current medical security status of the elderly in China. At present, there are few studies on the current situation of CHE among the elderly in China and the factors that influence it, and the use of panel data is rare. In addition, the introduction of Andersen’s model of health care utilization into the framework of this research allows for a more comprehensive study of the variables that influence CHE.

However, some limitations of this study should be acknowledged. First, our estimation of the proportion considered only incurred health costs, and the adverse impact of healthcare costs on households with member(s) who did not seek treatment because they could not afford it was not examined. Taking these omissions into account, the actual rate of physician use may be higher. Second, estimates of CHE in our study were influenced by the structure of the questionnaire, mode of data collection, and recall bias. However, these limitations do not invalidate our work, and the nature of large samples reduces estimation bias to some extent, as does the use of panel data.

## Conclusions

China’s health sector reform has achieved unprecedented progress, especially in terms of medical insurance coverage, but protecting the elderly from healthcare-related impoverishment remains a challenge. By examining the incidence and intensity of CHE and by identifying the main variables associated with CHE, several policy implications emerge from our study: (i) Poor households may abandon treatment, and the government can issue free medical vouchers for their use and promote their basic medical needs. Many countries are using this approach to narrow the health gap between different income groups [39, 40]. (ii) For households that already experience CHE, it is necessary to gradually improve medical assistance and ensure that their normal life is not affected by health expenditures. (iii) The function of health insurance to control the price of medical services needs to be more fully utilized. International research has also proven that investment in health insurance alone will not directly reduce out-of-pocket payments; measures such as the reform of payment methods to control the rapid growth of medical expenses also need to be strengthened [41].

## Data Availability

The datasets generated and analysed during the current study are available in the CHARLS repository, [http://charls.pku.edu.cn/en].

## Abbreviations

CHE: Catastrophic health expenditure
CHARLS: China Health and Retirement Longitudinal Study
O: Overshoot
MPO: Mean positive overshoot
NHCR: New Health Care Reform
UEBMI: Urban Employee Basic Medical Insurance
UBMI: Unified Basic Medical Insurance
NHSS: National Health Service Survey
OOP: Out-of-pocket healthcare payments
Hc: Headcount
